# Long-term care residents and staff perspectives on PARO using video-reflexive ethnography (VRE)

**DOI:** 10.3389/frdem.2026.1823277

**Published:** 2026-06-23

**Authors:** Yuka Ohno, Janna Zeid, Karen Lok Yi Wong, Lillian Hung

**Affiliations:** 1 IDEA Lab, School of Nursing, University of British Columbia, Vancouver, BC, Canada; 2 Faculty of Applied Science, School of Nursing, University of British Columbia, Vancouver, BC, Canada

**Keywords:** dementia, feasibility, implementation science, person-centered care, practice-based

## Abstract

**Background:**

Residents with dementia in long-term care (LTC) often experience social isolation, loneliness, and distress. Socially assistive robots such as PARO, have been introduced to support psychosocial well-being, yet prior studies have typically examined resident or staff perspectives separately, limiting understanding of how such technologies function within everyday care practice.

**Aim:**

This study examined the relational, practical, and organizational feasibility of using the social robot PARO in a special care unit by exploring both residents’ experiences and staff reflections.

**Methods:**

A qualitative ethnographic study was conducted in a Canadian LTC home. Residents living with dementia (*n* = 10) participated in group-based PARO sessions over 4 weeks. Video recordings, field notes, and conversational interviews captured residents’ experiences. Care staff (*n* = 10) engaged in video-reflexive ethnographic (VRE) group sessions using selected video footage to prompt discussion of benefits, barriers and implementation considerations. Data were analyzed using reflexive thematic analysis informed by person-centered care.

**Results:**

Feasibility emerged as relational, practical, and organizational. PARO promoted engagement and nurturing behaviors when actively facilitated by staff, but successful use depended on workflow integration, supervision capacity, infection-control procedures, and institutional resource priorities. VRE enabled staff to collectively identify implementation tensions grounded in observed interactions, reframing feasibility as a context-dependent process negotiated within daily care practice.

**Conclusion:**

The feasibility of social robots in dementia care depends on relational meaning, workflow integration, and organizational priorities. VRE is a valuable method for identifying implementation tensions early and supporting reflective, person-centered adoption of emerging technologies in LTC.

## Introduction

Older adults living with dementia in long-term care (LTC) frequently experience social isolation, loneliness, distress, and unmet psychosocial needs (Lapane et al., 2022; Koh et al., 2025; Boamah et al., 2021). These challenges are compounded by chronic staffing shortages, time pressures, and increasing care complexity, which limit opportunities for sustained social engagement and relational care (Chapman et al., 2024; Song et al., 2020). Within these constraints, staff often prioritize physical and task-based responsibilities, leaving fewer resources to address residents’ emotional and social well-being (Song et al., 2020). Identifying approaches that support psychosocial care while remaining workable within the realities of everyday LTC practice is therefore a pressing challenge in dementia care.

Socially assistive robots have emerged as one strategy to address these needs (Yu et al., 2022). The therapeutic robotic seal PARO, designed to provide companionship and emotional comfort, has been introduced in dementia care settings worldwide (Hung et al., 2019; Inoue et al., 2021). Studies report that interacting with PARO may increase social engagement, reduce distress, and improve mood and quality of life (Hung et al., 2019; Mervin et al., 2018; Pu et al., 2020; Chen et al., 2024; Shoesmith et al., 2024; Moyle et al., 2017). These findings suggest that social robots hold promise for enhancing psychosocial well-being in LTC environments. However, much of the existing literature focuses primarily on resident-level outcomes or staff attitudes (Yu et al., 2022; Rigaud et al., 2024). As a result, feasibility is often implicitly equated with effectiveness or acceptability. Less attention has been paid to how such technologies are negotiated within the relational, practical, and organizational conditions of everyday care. Recent ethnographic studies have shown how social robots can mediate care relationships, provoke ambivalence, reshape interactional dynamics, and become embedded within broader organizational and moral contexts of care (Persson et al., 2024; Chevallier, 2023; Ahlin and Mann, 2025). Building on this literature, the present study extends existing ethnographic work by combining participant observation with video-reflexive ethnography (VRE) to examine how residents’ embodied interactions with PARO inform staff deliberations about feasibility and implementation within LTC practice.

In practice, PARO does not function as a standalone intervention. Its use is mediated through interactions among residents, care staff, and the routines, constraints, and values of the care setting (Hung et al., 2019). Staff position the robot, facilitate engagement, interpret residents’ responses, ensure safety and infection control, and determine whether its use aligns with workflow demands (Hung et al., 2019; Koh et al., 2021). At the same time, residents’ engagement with PARO may influence staff’s perceptions of its value and appropriateness. Feasibility, therefore, cannot be determined solely by whether residents appear to benefit at the moment. It must also account for relational dynamics, workflow integration, and organizational priorities. Without such contextual insight, technologies that demonstrate promise in controlled studies may fail to translate into sustainable practice (Greenhalgh et al., 2017).

To address this gap, this study adopts video-reflexive ethnography (VRE) to examine feasibility as it unfolds *in situ*. VRE is a participatory, practice-based methodology that uses video recordings of everyday interactions to support collective reflection among practitioners (Iedema et al., 2022; Carroll et al., 2008; Ajjawi et al., 2020). Rather than relying solely on interviews or outcome measures, VRE renders tacit aspects of care visible and creates a dialogic space in which implementation tensions, practical constraints, and relational meanings can be surfaced and examined collaboratively (Iedema et al., 2018). In this study, VRE is used not merely as a data collection technique but as a method for identifying contextual barriers before implementation failure and for bridging resident experience with staff decision-making. By combining ethnographic observation of residents’ interactions with PARO and video-reflexive group discussions with care staff, this study conceptualizes feasibility as a relational, practical, and organizational achievement. Through this approach, we extend existing PARO research beyond individual-level effects and offer a context-sensitive account of how socially assistive robots are negotiated within everyday dementia care.

This study is guided by person-centered care (PCC) principles by Kitwood (1988). PCC emphasizes personhood as relational and dynamic, grounded in the fulfillment of fundamental psychosocial needs, including attachment, occupation, identity, comfort, and inclusion. Interactions that promote validation, collaboration, accessibility, playfulness, and relaxation support personhood, whereas interactions that undermine these needs diminish well-being. PCC provides an analytic lens for interpreting how PARO interactions support or challenge residents’ psychosocial needs and how staff evaluate the technology in relation to person-centered care. Kitwood’s (1988) framework informed interpretation of relational interactions during both resident observations and staff VRE discussions.

## Methods

### Research team

The research team consisted of two research trainees, seven undergraduate volunteers, and an academic supervisor, totaling ten people. Authors YO and JZ are female research trainees with a cognitive science background. The trainees and research assistants received training in research coordination, observational data collection, and facilitation of interactive sessions under LH’s supervision. The team had no prior relationships with participants before the study commenced.

### Study design

This study employed a qualitative ethnographic design incorporating video-reflexive ethnography (VRE) to examine the feasibility of using the social robot PARO in a LTC special care unit. This approach aligns with ethnographic traditions in human–robot interaction research that examine robots within situated care practices rather than controlled laboratory environments (Felding et al., 2024). The design included: (a) ethnographic observation of residents’ interactions with PARO and (b) video-reflexive group discussions with care staff. This approach enabled feasibility to be examined as a relational, practice-based process, attending to how PARO was experienced, interpreted, and negotiated within everyday care routines and organizational constraints.

### Setting and participants

Our study was conducted at an urban publicly funded LTC home in British Columbia. The special care unit accepts residents requiring medical and nursing care specifically for symptoms of dementia. The unit care team is trained to work with older adults living with dementia, offering perpetual, person-centered care, including physicians, registered nurses, licensed practical nurses, certified care aides, therapy staff, and social workers. The special care unit provides 36 beds, and all rooms are private.

Ten resident participants (Table 1a) were identified through selective sampling with the assistance of an LTC educator. The inclusion criteria for resident participants were: (1) residing in the LTC home; (2) having a diagnosis of dementia at any stage; and (3) being able to communicate in English. Some participants were more fluent in verbal expression, while others experienced greater cognitive and communication challenges in social interactions. The recruited participants were 6 females and 4 males, who had a diagnosis of dementia at various stages and other co-morbidities. Individuals who had communication and/or mobility difficulties were not excluded from the study. Due to the nature of dementia symptoms, some resident participants experienced limitations in providing full informed consent, and their family members were invited to provide consent to participate in the study on their behalf. Assent from the residents was sought for study participation, and their verbal or behavioral dissent was respected (Black et al., 2010).

**Table 1a:** Resident participant characteristics.

Characteristics	*N* (%) (*n* = 10)
Age	
60–74	4
75-	6
Gender	
Female	6
Male	4
Ethnicity	
Asian	3
White	7
Mobility	
Wheelchair	7
Walker	2
In-Bed	1

Ten staff members (Table 1b) were recruited through convenience sampling. The inclusion criteria for staff were: (1) working on-site in the same special care unit the residents reside in; and (2) willingness in participation. They were invited to offer their clinical expertise from working in a care setting, as well as to provide interpretations of the residents’ interactions with social robot PARO because many older adult residents had language limitations.

**Table 1b:** Care staff participant characteristics.

Characteristics	*N* (%) (*n* = 10)
Age	
20–30	1
31–40	5
41–50	4
Gender	
Female	8
Male	2
Ethnicity	
Asian	5
White	5
Position	
Manager	1
Recreational staff	4
Nurse(s)	1
Care aides	4

### Procedure

#### Robot interactive sessions

An informational meeting was held prior to recruitment to introduce PARO and explain study procedures to staff. Residents participated in group-based interactive sessions twice weekly over four consecutive weeks (February–March 2025). Each session lasted approximately 40 min. During sessions, residents took turns interacting with PARO while others observed. Residents were free to engage as they wished—hugging, stroking, speaking to the robot, or observing from a distance. Individual engagement ranged from approximately 5–10 min per session. One staff member was present during each session to provide care support. Consent was treated as an ongoing process (Black et al., 2010). Verbal assent was obtained at the start of each session and revisited as PARO rotated among residents. Any verbal or behavioral indication of discomfort resulted in immediate withdrawal from interaction (see Table 2).

**Table 2:** Example thematic analysis.

Themes	Codes	Quotations
PARO is a resource when it engages residents	Resource; engagement; visible happiness; shared	*“PARO definitely brought smiles to our residents. They were able to talk about their experiences with one another… a shared opportunity that fosters conversations. I could also see that if a resident has that connection to PARO, it might be a great resource for pain management or end of life support. It’s a resource that we can engage the residents with.”*
PARO would be efficient if it aligns with workflow	supervision Operations; technical; infection control; constant	*“We want something that… frees up a little bit of time for the staff to focus on something else. PARO is easy to use, but it was heavy for frail residents in wheelchairs. We are also worried that the residents will feed PARO. If it has to be supervised all the time… it probably will not be successful.”*
PARO is justifiable if it outscores existing options	Adaptable; comparison; budget;	*“If we can have PARO, we will use it. But the residents like the stuffies just as much. We have a doll therapy program and robotic cats that simulate heartbeat, and they work well… PARO is kind of expensive, too. A high-functional robot is not really needed.”*

#### Video-reflexive ethnography (VRE) sessions

Following completion of the four-week interactive period, two 30-min VRE group sessions were conducted: one with day-shift staff (*n* = 5) and one with night-shift staff (*n* = 5). Splitting sessions minimized disruption to care routines and encouraged participation. The purpose of VRE sessions was to examine feasibility within everyday practice. Staff viewed five short video clips (each under 20 s) of resident–PARO interactions selected to represent variation in gender, communication style, and engagement patterns. Clips included residents demonstrating initial hesitation, sustained connection, observational participation, and evolving responses over time. Footage was not used to evaluate performance, but to surface tacit knowledge about relational dynamics, workflow integration, safety considerations, and organizational constraints. Staff were asked:

- What do you notice in these interactions?
- Under what conditions might PARO be useful?
- What barriers might arise if PARO were integrated into routine care?
- How might it align (or not) with the current workflow?

Both sessions were audio-recorded with consent.

VRE sessions were guided by principles of exnovation, collaboration, reflexivity, and care (Iedema et al., 2018). Exnovation makes the everyday, taken-for-granted practices visible through video to reveal tacit care knowledge and hidden complexities. Collaboration positions practitioners as co-analysts who actively contribute to interpretation and improvement. Reflexivity enables participants to critically review and interpret assumptions of care practices and reconsider the habitual ways of care delivery. Care ensures a respectful environment where researchers act as facilitators rather than evaluators, fostering a psychologically safe environment focused on collective learning rather than critique (Iedema et al., 2018).

### Data collection

Multiple qualitative data sources were used to capture resident experiences and staff reflections on the feasibility of integrating PARO into routine care.

During interactive sessions, conversational interviews were conducted to explore residents’ experiences with PARO. Open-ended prompts such as “What do you think about PARO?” and “How does it make you feel?” invited residents to describe their emotional and social responses. Follow-up questions were used to clarify meaning and deepen understanding (e.g., “What do you mean by ‘It’s a treat for me’?”). These exchanges often elicited personal stories, memories, and spontaneous reflections, providing insight into residents’ intuitive and embodied responses to the robot.

Video ethnography was used to capture subtle interactional details, including facial expressions, gestures, posture, gaze, and touch. The research team used an iPhone 14 Pro to record these observations with consents, simultaneous to other student volunteers taking observational field notes. This ethnographic observation took place when the participant agreed to be filmed, during which the participant interacted with PARO for 5–10 min. Observational field notes documented verbal statements and social dynamics during sessions. Residents were given the opportunity to review recorded footage and confirm consent for its use in analysis. The video footages were exported to the PI’s cloud drive and were deleted from the iPhone after the sessions ended.

Audio recording was employed to collect the staff VRE group sessions. Researchers acted as facilitators rather than evaluators, positioning staff as holders of contextual expertise. Discussion emphasized collective learning and improvement rather than critique of individual practice. The environment was intentionally non-judgmental and psychologically safe to support open dialogue. Through this process, staff articulated relational meanings, workflow considerations, supervision demands, infection-control implications, and organizational trade-offs influencing feasibility. Both VRE session participants gave consent to audio recordings for subsequent thematic analysis.

### Data analysis

Video-recordings of residents’ interactions were transcribed verbatim, and non-verbal expressions such as smiling, kissing, and caressing the social robot were added to the script. Observational field notes were compiled alongside transcripts and organized using direct quotations with pseudonyms to maintain contextual richness. Resident data were initially reviewed and analyzed to inform the selection of video clips for the video-reflexive ethnography (VRE) sessions. Following preliminary coding of all recorded sessions, five clips were purposively selected to represent variation in resident gender, communication style (verbal and non-verbal), and engagement patterns (e.g., initial hesitation, sustained interaction, observational participation). The aim was not to highlight optimal or particularly successful interactions, but to capture a range of relational dynamics and practical considerations that could meaningfully stimulate discussion about implementation feasibility. These selected clips were shown to staff during VRE group sessions to prompt collective reflection. Audio recordings of the staff discussions were subsequently transcribed verbatim for analysis. Reflexive thematic analysis was conducted across both resident and staff datasets in alignment with Braun and Clarke (2006). Transcripts were coded iteratively, with codes grouped into themes addressing the study aim. Analysis combined inductive coding grounded in participants’ accounts with deductive attention to existing literature on person-centered care and implementation feasibility. Together, this analytic approach enabled the identification of relational, practical, and organizational dimensions of feasibility as articulated through the VRE sessions.

### Researcher positionality

The research team approached this study from a person-centered, relational-care perspective, drawing on prior experience in dementia care research and qualitative inquiry. Researchers were not detached observers but were actively engaged in facilitating PARO interactions and building rapport with residents during sessions. This positioning shaped both the interactions that emerged and the meanings interpreted from them. Rather than aiming for observational neutrality, the study acknowledged that resident engagement with PARO was co-constructed through researchers’ presence, facilitation, and relational responsiveness. Reflexive discussions were conducted throughout data collection and analysis to critically consider how researchers’ assumptions, interactions, and interpretations may have influenced the data generated.

### Ethical considerations

This study was reviewed and approved by the University Research Ethics Board (H24-03599). Study participants and their families were fully informed about the study, including the research purpose and their rights to participate and withdraw from the study at any time. Due to the nature of dementia, some residents were not able to sign the consent form. In such circumstances, their family member signed the participation information and initial consent on their behalf. This consent process was approved by the Ethics Board in a situation where the participants are unable to sign informed consent due to their medical conditions. All the study data are collected with the participants’ consents and assents, and any field notes and video footages collected are made anonymous for confidentiality.

In the consent form, the participants were given the option for the researchers to film and use video recordings for data analysis purposes. The researchers showed the video footage during the interactive sessions. Some participants preferred that the researchers take observational field notes instead of video and audio recordings. All participants permitted the research team to use emerging data in direct quotes for publication and conference presentations. Data is de-identified and processed confidentially, and pseudonyms are used to protect participants’ identities. The social robot PARO has an internal mic and artificial intelligence chip installed for audio detection. Its purpose is to gauge the emotional tone and environmental context and generate appropriate responses, and it does not store the conversation. The audio data is processed internally to the robot and then discarded, without being uploaded and stored externally.

## Findings

Reflexive thematic analysis generated three interrelated themes: (1) PARO is a resource when it engages residents; (2) PARO would be efficient if it aligns with workflow; and (3) PARO is justifiable if it outscores existing options. Together, these findings illustrate that feasibility extends beyond resident enjoyment to encompass contextual and strategic considerations within LTC.

### Theme 1: PARO is a resource when it engages residents—relational feasibility

Relational feasibility emerged when PARO facilitated meaningful engagement that staff recognized as aligned with person-centered care. Across interactive sessions, residents demonstrated sustained tactile contact, focused gaze, verbal expression, and affective responsiveness. These embodied behaviors were interpreted by staff as indicators of connection and emotional presence. Across the video excerpts, the residents consistently showed visible engagement with the social robot, including smiling, leaning forward, maintaining prolonged touch, sustaining eye contact with and talking to the robotic seal. Residents attempted to pull PARO nearby, held it in their arms or on the lap, and kissed or stroked its fur. A recreational care staff, Nancy, was pleased to see residents’ joyful interaction and shared that “the residents look happy.” Another care staff, Lora, agreed that “it definitely brought smile to our residents.” Nancy continued, describing PARO as “irresistible,” noting its advanced sensory and interactive feature “sparked joy” among residents. It was an outcome aligned with what staff “wanted to accomplish.” Visible happiness among residents shaped staff’s acceptance of PARO as a care resource on the unit. Across both VRE sessions, staff returned to residents’ visible engagement as central to judge its value.

However, staff reflections also revealed that PARO’s relational impact did not occur independently but enabled through active facilitation. Engagement was sustained when staff positioned PARO within reach, guided residents’ hands toward it, narrated its sounds or movements, and invited verbal responses. Staff often personalized the interaction, framing the robot as a responsive agent (e.g., “He likes you,” “She’s talking to you”). Through these guided strategies, residents appeared to be more engaged with the robot. Without active facilitation, staff anticipated that the robot could become a mere object “sitting in the corner of the room.” Relational feasibility, therefore, emerged through structured, intentional involvement rather than through the robot’s interactive features alone.

In group settings, staff described that PARO mediated relationships among residents. Care staff Lora reflected that carrying out “social conversations on the unit was rare,” as residents living with mild to moderate dementia often struggle to connect with one another due to challenges with memory loss or “lack of a shared intimate experience.” PARO created a common point of attention and offered an opportunity for residents to converse about. Staff noticed that they initiated more peer-to-peer conversations and sometimes monitored turn-taking to ensure equal participation. Patricia, a recreational care staff, described that PARO created a “meaningful opportunity to care for someone,” as it enabled residents to temporarily restore nurturing roles instead of always being taken care of. Shelly, a manager, agreed that this caring behavior aligned with how the care team evaluated residents’ wellness.

Observational field notes indicated that seven out of the ten resident participants consistently engaged with PARO directly, while the remaining three preferred indirect participation. Even those who did not physically interact remained in the group voluntarily, observed others, and occasionally initiated conversation. For instance, a male resident with some verbal limitation, Patrick, did not wish to further interact with PARO after his initial try-out. Instead, he preferred to take part from a distance, by seeing others engage. He said that he “just [wanted] to see others” (verb present tense changed to past tense) engaging with PARO. When the study team asked him whether he liked PARO, he agreed and gave a reason: “it makes me happy.” Staff interpreted that his decision to stay was probably influenced by a sense of belonging to this PARO interaction group.

Beyond group context, staff contemplated individualized use of PARO in pain management and palliative care. Many discussed that PARO’s tactile and sensory qualities could be tailored to a person’s comforting needs. For example, Amy, a therapeutic care staff, thought about using PARO as a “positive distractor” for residents in pain management. Through VRE, she saw that resident Ashley would likely benefit from this approach, as she noticed her strong connection with the robot. One time, she invited Ashley for her previously favorite teatime, but she unexpectedly refused and told Amy that she wanted to stay with PARO. When the study team asked her why she liked PARO, she said “it’s very gentle and kind.” Noting this, Amy addressed that PARO was “something different,” implying that it could be effective at soothing responsive behaviors for residents like Ashely.

### Theme 2: PARO would be efficient if it aligns with workflow—practical feasibility

Staff emphasized that a care tool would most likely be used when it provides care benefits that align with workflow. Chronic understaffing, time pressures, and growing care complexity pose significant challenges for staff to balance responsibilities while maintaining the quality of care. Therefore, one desired care outcome here was to improve operational efficiency. The manager, Shelly, told the study team that “We want something that engages residents and frees up a little bit of time for the staff to focus on something else.”

However, multiple staff reported that delivering care using PARO requires constant supervision regardless. Unlike teddy bears or crayon kits available in the unit, it could not be simply handed over due to concerns with accessibility, safety, and ongoing maintenance requirements. Through VRE, staff noticed that the weight and size of PARO could be challenging for some residents in wheelchairs. These individuals struggled to hold the robot independently because of limited upper-body strength or because the robot was too wide to be placed comfortably in wheelchairs. In these cases, the interaction required staff to actively support placement and reorientation of residents, such as holding the robot nearby, adjusting residents’ posture, stabilizing PARO on their lap or repositioning the robot on a nearby surface, so that it stayed within reach. The effectiveness of the robot depended on these technical positioning and secure interactions without creating risk of dropping, discomfort, or frustration. As a result, manager Shelly commented, “It’s soft and cute and everything, but I don’t know,” implying her uncertainty regarding PARO’s efficiency in desired care usage. Safety was another consideration. A recreational care staff, Patricia, recalled that residents had previously attempted to feed another robotic animal. She noted that “residents want to feed the dolls… and that’s sort of an issue.” Staff expressed similar concerns about PARO and interpreted a demand for monitoring. Besides accessibility and safety conditions, staff discussed ongoing maintenance tasks, including infection-control routines (e.g., cleaning using disinfection wipes, sanitizing residents’ hands, etc.), ensuring PARO is charged and ready for use, and storing it appropriately when not in use. These procedures outside of the care intervention were deemed added tasks to frontline staff. Manager Shelly, clarified that while the robot itself was not difficult to operate, if a tool is not always ready for immediate use, then the implementation “probably will not be successful.” However, she added that it could be workable providing logistical challenges could be overcome.

Overall, staff characterized practical feasibility as conditional and context dependent. Resources in LTC are adopted not only because “they are easy to use,” but because they can be “accessed quickly to engage a resident.” Practical feasibility therefore extended beyond whether PARO functioned as designed. It depended on whether it could be reliably integrated into the pace, staffing realities, and workflow of a busy unit without adding unintended complexity.

### Theme 3: PARO is justifiable if it outscores existing options—organizational feasibility

Organizational feasibility was determined by whether PARO offered meaningful, added benefits beyond what was already available on the unit. Existing care resources included a doll therapy program and robotic cats that could simulate heartbeat. According to Shelly, these existing care tools were therapeutic for residents as it could “mold into residents’ bodies for comfort.” Given PARO’s accessibility concerns for frail residents, she pointed out that “PARO does not mold into body” for comfort as intended. In addition, she reflected that residents enjoy existing resources “just as much,” suggesting the comforting needs can be sufficiently addressed by available options. While recognizing PARO’s adaptable potential in both group and individual settings, staff were worried that its lack of liveliness (e.g., heartbeat, warmth) beyond interactivity could diminish care outcomes over time. Building on this comparison, some staff questioned the necessity of introducing a high-functioning robot into an environment where simpler alternatives were already effective. Care staff Nancy noted that sensory qualities such as softness or warmth were sufficient calming characteristics for most residents on the unit. As such, staff perceived PARO’s added value as incremental rather than transformative within the current care environment.

Sourcing new resources would require integration of infection-control procedures, charging routines, safe storage, and consistent supervision into daily workflow. Staff considered who would assume responsibilities for these tasks and where the robot could possibly be stored. Manager, Shelly, surfaced a possible routine in which PARO could be kept in a visible “basket in the corner” and cleaned using disinfection wipes before use. She further considered that charging and maintenance details could work out if planned carefully. Organizational feasibility was therefore connected to resource trade-offs. Time spent setting up, maintaining, and supervising PARO could potentially reduce time available for other programming or person-centered care activities. In a unit with already balancing multiple demands, introducing a new tool requires careful consideration of how it would fit within existing responsibilities. As Shelly noted, resources are not adopted simply because they function well, but because they can be accessed and sustained reliably within the flow of the unit.

At the broader level, sustainability beyond structured sessions was another point of consideration for PARO adoption. Care staff Lora expressed uncertainty about whether residents’ engagement with PARO would endure beyond a novelty effect. She emphasized the importance of ensuring that innovative care tools can be used sustainably, particularly as artificial intelligence-driven devices become more common in care. Given that PARO’s current market cost is estimated at USD 6,000, the financial commitment also shaped organizational deliberation. As existing alternatives were cost-efficient (a couple hundred dollars, according to staff) and already meeting residents’ needs, Shelly concluded that “a high functional robot is not really needed” at the moment. Overall, successful implementation would require justification on necessity and priorities within financial and operational capacity. In this context, feasibility was extended to including whether PARO offered a sustainable and strategically appropriate investment within the existing care ecology.

## Discussion

Introducing PARO into the special care unit required more than acquiring a device; it required examining whether the technology could be delivered reliably, safely, and sustainably within the realities of LTC. In the study, feasibility did not emerge as a simple question of effectiveness (“Does PARO work?”), but as a contextual and relational question (“Can PARO work here, under these conditions?”). By integrating resident interactions with staff video-reflexive discussions, this study reconceptualizes feasibility as a situated accomplishment shaped by relational meaning, workflow integration, and organizational priorities.

### Feasibility as relational

Residents demonstrated visible joy, engagement, and nurturing behaviors when interacting with PARO. These embodied responses, smiling, stroking, sustained gaze, and reluctance to disengage, signaled meaningful connection. However, such engagement did not occur independently of staff involvement. Interactions were scaffolded through positioning, narration, encouragement, and monitoring. For instance, staff’s deep appreciation of each resident’s interests and abilities enabled them to anticipate interactions with PARO as positive distractors to mitigate distress, and to foresee potentially problematic interactions that warrant practical considerations (e.g., residents attempting to feed PARO). Staff enacted Kitwood’s (1988) personhood-promoting facilitation to guide and encourage residents’ mode of interaction with PARO (e.g., as a conversation prompt, tool for turn-taking, or a sensory stimulus for less verbal residents) and consider when other resources or distractors would fit more. Conversely, by allowing residents’ choices in their engagement with PARO with no expectations and letting interactions organically emerge, staff enacted Kitwood’s (1988) personhood-promoting collaboration, as well as play and celebration. By actively interpreting residents’ cues for preferred engagement method with PARO, staff were able to facilitate meaningful interactions, and, reciprocally, residents’ organic interactions with PARO enabled spontaneous connections with staff. Thus, PARO functioned less as an autonomous therapeutic tool and more as a relational mediator embedded within staff–resident interaction, which overlapped with Kitwood’s (1988) PCC. This finding aligns with ethnographic studies describing social robots as relational and socially negotiated artifacts whose meanings emerge through interactions among residents, staff, and care environments rather than through technological features alone (Persson et al., 2024; Chevallier, 2023). Feasibility at the relational level, therefore, depended on the capacity of staff to intentionally facilitate engagement. Without active mediation, the robot risked becoming a passive object rather than a catalyst for connection. These findings challenge technological narratives that implicitly frame social robots as substitutes for human presence (Sharkey and Sharkey, 2012). As such, the robot’s value was not inherent but co-constructed within everyday care practices.

Moreover, staff interpreted residents’ embodied responses in light of their broader knowledge of individual histories and personalities. A sustained touch or refusal to leave PARO for another activity was not read as novelty alone, but as potentially meaningful comfort, attachment, or identity expression. Through VRE, these subtle moments became visible and discussable, allowing relational meaning to inform deliberation about future use. Residents’ nurturing behaviors toward PARO may reflect Kitwood’s (1988) psychosocial needs of attachment, occupation, identity, and inclusion. The robot created opportunities for residents not only to receive care, but also to enact caring roles, reinforcing personhood through relational engagement. Rather than functioning solely as an individual therapeutic device, PARO also became a shared relational object that mediated interaction among residents and staff. This aligns with Kitwood’s (1988) view that personhood emerges relationally through social interaction rather than residing solely within the individual.

### Feasibility as practical

While staff recognized relational value, they simultaneously identified supervision demands, infection-control procedures, charging requirements, physical weight and positioning challenges, and workflow alignment as critical constraints. Importantly, these concerns were grounded in observed interactions rather than abstract speculation. For example, staff noted that frail residents struggled with PARO’s size and weight, raising questions about accessibility and safe positioning before any large-scale adoption. These practical reflections illuminate a common implementation tension: technologies often demonstrate functional promise under structured or researcher-facilitated conditions but encounter friction within routine practice (Koh et al., 2021; Greenhalgh et al., 2017). LTC environments operate under time pressures, staffing constraints, and competing responsibilities. A tool’s adoption depends not only on whether it is effective, but on whether it can be accessed quickly, integrated seamlessly, and maintained without introducing additional burden (Rigaud et al., 2024). Our findings suggest that positive resident responses alone are insufficient to determine feasibility. Even when residents appeared calm and engaged, staff questioned whether constant supervision or maintenance demands would be sustainable. Feasibility, therefore, extended beyond usability to encompass workflow compatibility and temporal fit within a busy unit. Staff reflections also suggested that worthwhile interventions in LTC may require organizational adaptation rather than immediate efficiency gains. Some participants viewed the additional time needed for PARO facilitation as potentially justified if the intervention supported meaningful social connection and emotional comfort that residents may not otherwise experience. This highlights a broader tension in LTC between operational efficiency and relational care, where interventions that require time and facilitation may still be valued when they contribute meaningfully to residents’ psychosocial well-being. In this way, practical feasibility functioned as a threshold condition for sustained integration.

### Feasibility as organizational

Beyond relational and practical considerations, staff framed feasibility in terms of organizational trade-offs and resource allocation. PARO was evaluated alongside existing alternatives, including doll therapy and robotic cats. Staff weighed its added value against cost, storage requirements, infection-control integration, and charging routines. The question was not whether PARO could work in isolated moments, but whether it justified investment relative to existing tools that were already embedded within practice. Feasibility thus became an organizational deliberation about priority and sustainability. Adoption required alignment not only with resident needs and workflow, but also with financial constraints and strategic direction. Staff discussions reflected broader organizational tensions common in LTC, where innovation must compete with staffing shortages, infection-control demands, and resource limitations. Feasibility was therefore shaped not only by the technology itself, but by institutional cultures that prioritize interventions perceived as sustainable, low burden, and operationally compatible. This broader perspective situates social robots within a care ecology rather than as standalone solutions (Koh et al., 2021). In complex systems such as LTC, innovations should compete with established practices for legitimacy, time, and resources. Similar ethnographic work has demonstrated that robotic technologies in care settings are often shaped by institutional routines, competing priorities, and moral negotiations surrounding care work and resource allocation (Felding et al., 2024).

### The methodological contribution of VRE

Video-reflexive ethnography was central to uncovering these multi-level dimensions of feasibility. By replaying resident–PARO interactions, VRE made subtle aspects of practice visible and discussable. Moments that might otherwise have remained fleeting or individually interpreted were collectively examined. In this sense, VRE functioned as a way of identifying barriers before implementation failure. Rather than assuming adoption based on promising outcomes, the method supported identifying contextual tensions while adaptation remained possible.

Importantly, VRE did not resolve barriers; it rendered them visible. By doing so, it acted as a methodological safeguard against premature or top-down technology adoption detached from everyday care realities. Technologies in healthcare frequently fail not because they lack benefit, but because relational and organizational conditions are insufficiently examined before implementation (Koh et al., 2021; Greenhalgh et al., 2017). VRE enabled these conditions to be surfaced prior to institutional commitment. Beyond identifying barriers, VRE operates as a mechanism for collective sense-making. Staff drew on their contextual expertise to interpret what they observed in the video clips. Through dialogue, they negotiated the significance of resident responses: Was PARO calming? For whom? Under what circumstances? Could it support distress management or serve as a comforting presence in palliative contexts? Feasibility shifted from being a technical judgment about device performance to a shared organizational inquiry grounded in lived practice.

VRE also bridged resident experience and staff decision-making. For residents living with dementia, preferences and meanings are often expressed through embodied responses rather than sustained verbal articulation. Video preserved these subtle gestures, touch, posture, gaze, and rendered them durable for reflection. In doing so, VRE reduced the epistemic distance between lived experience and implementation deliberation. Residents’ embodied expressions were incorporated into organizational discussion, aligning adoption decisions with person-centered care principles. At the same time, it is important to acknowledge that VRE sessions, like most group-based methods, can be influenced by power dynamics. In our study, most of the presenting findings originated from certain staff participants (i.e., manager, care aides and other recreational staff). Hierarchical structures within healthcare teams may influence whose voices are amplified and whose concerns are muted (Essex et al., 2023). Although facilitation aimed to foster psychological safety and collaborative dialogue, collective reflection does not eliminate organizational power relations. Future studies might be interested in understanding such hierarchical dynamics of group reflections in VRE.

These findings contribute to implementation science by reframing feasibility as a situated, negotiated accomplishment rather than a preliminary checkbox preceding adoption. In complex care environments, technologies do not succeed solely based on demonstrated effectiveness (Koh et al., 2021). They must align with relational practices, operational rhythms, and institutional priorities. Failure to examine these dimensions early may lead to downstream implementation challenges or abandonment (Pourazad et al., 2026). Embedding reflexive evaluation methods such as VRE into early-stage innovation processes may thus support context-sensitive adoption. By making practice visible and fostering collaborative deliberation, VRE enables care teams to anticipate barriers, negotiate contextual fit, and adapt strategies before significant financial or cultural investment occurs. In this way, feasibility becomes not a static assessment, but an ongoing process of alignment between technology and the care environment. While previous ethnographic studies have primarily examined everyday interactions around social robots, our study extends this work by integrating video-reflexive group discussions with staff to examine how observed resident interactions become translated into organizational judgments about feasibility, workflow integration, and implementation.

## Strengths and limitations

A key strength of this study is its methodological integration of ethnographic observation with video-reflexive group discussion, enabling multi-level analysis of feasibility. However, several limitations should be noted. The study was conducted at a single site with a small sample, limiting the transferability of the findings. The four-week duration restricts understanding of long-term sustainability and potential novelty effects. PARO was not accessible to staff outside structured sessions, limiting opportunities for spontaneous use and longer-term program development. Family members of the resident participants were not included in this study for data analysis, which may have limited exploration of the resident’s interpretations on their experiences in this study. Additionally, VRE is time-intensive and requires trust-building, iterative analysis, and careful facilitation, which may present challenges for broader implementation.

## Conclusion

This study demonstrates that the feasibility of integrating social robots into dementia care is not determined solely by resident enjoyment or technological sophistication, but through ongoing relational and organizational negotiation within everyday care practice. By combining residents’ embodied interactions with PARO and staff video-reflexive discussions, this study reconceptualizes feasibility as a situated accomplishment shaped by relational meaning, workflow integration, institutional priorities, and care values. While PARO facilitated meaningful moments of connection and nurturing behaviors, its sustained adoption depended on supervision capacity, accessibility, infection-control routines, workflow compatibility, and resource allocation within the LTC environment. Methodologically, this study extends ethnographic research on social robots by demonstrating how VRE can support implementation inquiry in dementia care. By making everyday practice visible and supporting collective reflection, VRE surfaced implementation tensions before large-scale adoption and bridged residents’ embodied experiences with staff organizational decision-making. Future implementation of socially assistive technologies in LTC should therefore incorporate reflexive, practice-based approaches that attend to the relational, ethical, and organizational conditions shaping sustainable person-centered care.

## Data Availability

The raw data supporting the conclusions of this article will be made available by the authors, without undue reservation.
